# MiR-202-5p is a novel germ plasm-specific microRNA in zebrafish

**DOI:** 10.1038/s41598-017-07675-x

**Published:** 2017-08-01

**Authors:** Jing Zhang, Wei Liu, Yilin Jin, Peng Jia, Kuntong Jia, Meisheng Yi

**Affiliations:** Guangdong Provincial Key Laboratory of Marine Resources and Coastal Engineering, Zhuhai Key Laboratory of Marine Bioresources and Environment, School of Marine Sciences, Sun Yat-sen University, Guangdong, China

## Abstract

Gametogenesis is a complicated biological process by which sperm and egg are produced for genetic transmission between generations. In many animals, the germline is segregated from the somatic lineage in early embryonic development through the specification of primordial germ cells (PGCs), the precursors of gametes for reproduction and fertility. In some species, such as fruit fly and zebrafish, PGCs are determined by the maternally provided germ plasm which contains various RNAs and proteins. Here, we identified a germ plasm/PGC-specific microRNA miR-202-5p for the first time in zebrafish. MiR-202-5p was specifically expressed in gonad. In female, it was expressed and accumulated in oocytes during oogenesis. Quantitative reverse transcription PCR and whole mount *in situ* hybridization results indicated that miR-202-5p exhibited a typical germ plasm /PGC-specific expression pattern throughout embryogenesis, which was consistent with that of the PGC marker *vasa*, indicating that miR-202-5p was a component of germ plasm and a potential PGC marker in zebrafish. Our present study might be served as a foundation for further investigating the regulative roles of miRNAs in germ plasm formation and PGC development in zebrafish and other teleost.

## Introduction

Primordial germ cells (PGCs), the progenitor cells of gametes, play critical roles in genetic information transmission from generation to generation^1^. In most animals, PGCs segregate from somatic cells at early developmental stages and migrate to genital ridge where PGCs and gonadal somatic precursors together form primordial gonad^2^. Germ plasm is an amorphous, non-membrane-bound, electron-dense structure comprising of abundant RNAs, proteins and organelles, which is essential for PGC development in most animals^3–5^. By presence or absence of maternal germ plasm, two modes of PGC specification, preformation and induction, are proposed in animals^6^. In worms, flies and zebrafish, PGC specification is dependent on the maternal germ plasm accumulated in eggs (preformation)^7^. In urodele amphibians and mammals, extracellular signals induce PGC specification during gastrulation (induction). Many components of germ plasm have been revealed to play important roles in PGC development. The RNA binding protein Vasa regulates localization of another germ plasm component *nanos* which mediated transcriptional repression is critical for PGC maintenance^8^. Daz, Dazl and Boule are three key factors in regulating germ cells entering meiosis^9, 10^. Dnd protects critical germ plasm mRNA from degradation in germ cells by inhibiting miR-430^11, 12^.

MicroRNAs (miRNAs) are about 22 nucleotide endogenous non-coding RNAs that play important roles in posttranscriptional regulation by targeting mRNA for degradation or translational repression^13^. Recently, it has been reported that miRNAs play an important regulatory role in germ cell proliferation, specification, migration and maintenance in flies and mammals^14–16^. Targeted depletion of the miRNA-processing enzyme Dicer1 in germ cell of worms, flies and mice led to PGC reduction and gametogenesis defects^17–20^. In chicken, microarray assay revealed some germ cell-enriched miRNAs, suggesting that miRNAs might regulate germ cell development^21^. In human, it had been found that a miR-372/let-7 axis regulated germ cell maintenance and differentiation^22^. In zebrafish, little is known about the role of miRNAs in germ cell development, and it has been remained uncertain whether there are germ plasm/PGC-specific miRNAs in zebrafish.

MiR-202–5p is a miRNA abundantly expressed in gonad of some mammals and lower vertebrates. In mouse, miR-202-5p is specifically expressed in Sertoli cells of embryonic gonads^23^. In human, the Sertoli cell-specific miR-202-5p might play an interaction role between somatic cells and germ cells in spermatogenesis^24^. In frog, miR-202-5p is a germ cell-enriched miRNA in testis and ovary, indicating miR-202-5p might also be involved in germ cell development in amphibians^25, 26^. Recently, it has been reported that miR-202-5p and miR-202-3p are expressed throughout gonad development in zebrafish by transcriptomic analysis of miRNA expression in gonads^27^. Previously we reported that miR-202-5p was highly expressed in spermatozoa and developing male germ cells in zebrafish^28^. However, the expression patterns of miR-202-5p and -3p are remained unknown in embryos. In this study, the expression patterns of miR-202-5p and -3p were characterized in tissues and embryos, and found that miR-202-5p rather than miR-202-3p was highly and specifically expressed in gonads of zebrafish. Moreover, whole mount *in situ* hybridization (WISH) and fluorescence *in situ* hybridization (FISH) revealed that miR-202-5p was a maternal component of germ plasm and was specifically expressed in PGCs throughout embryogenesis. Therefore, for the first time, we identified a germ plasm-specific miRNA miR-202-5p in zebrafish, and it might be a potential marker for PGCs.

## Results

### Gonad-specific expression of miR-202-5p in zebrafish

**Figure 1:**
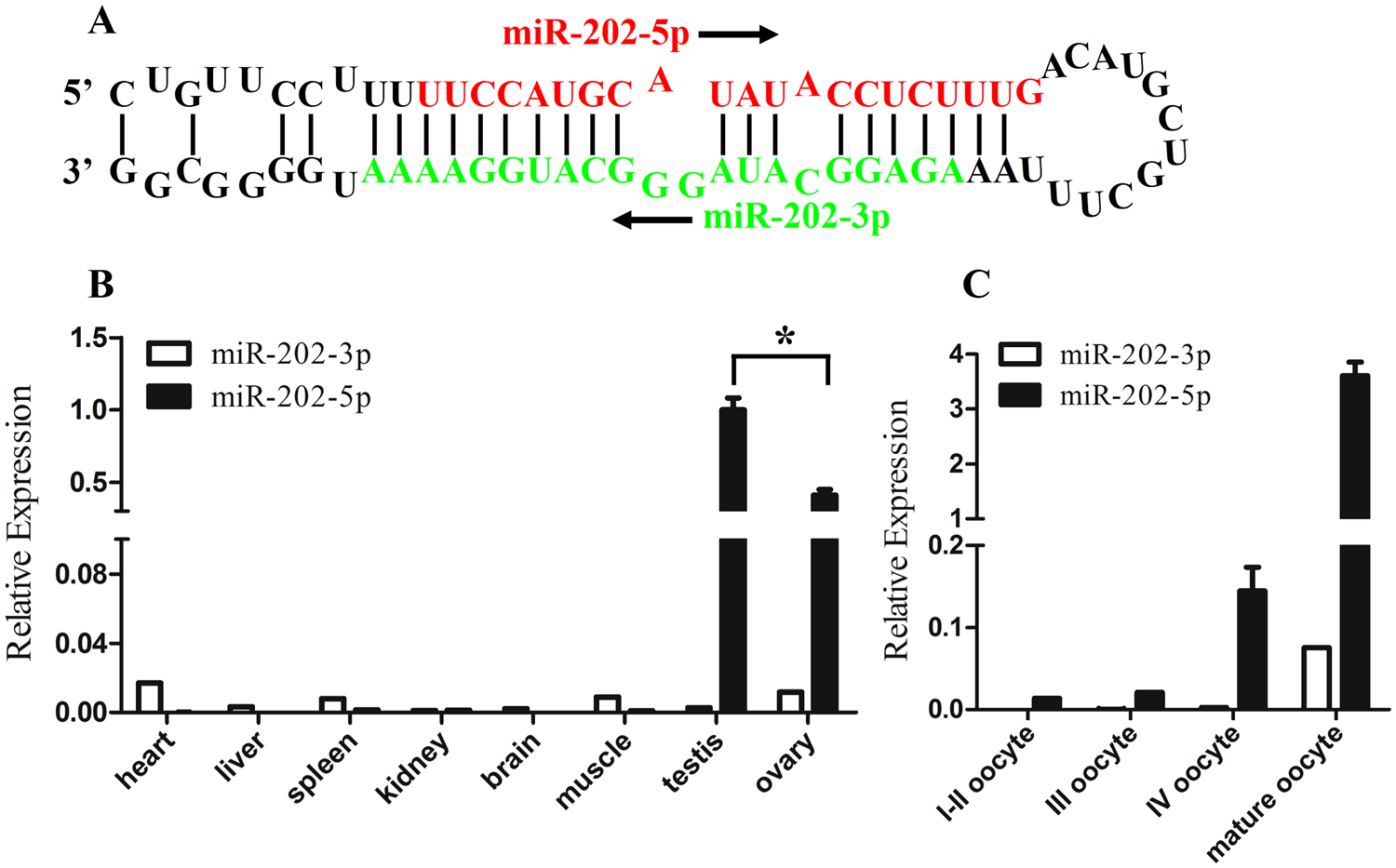
Tissue distribution pattern of miR-202 by RT-PCR. (**A**) Sequence of miR-202 including stem loop, miR-202-5p (in red) and miR-202-3p (in green). The arrow indicated the direction of miRNA sequences from 5′-3′. (**B**) Tissue distribution of miR-202-3p/5p. (**C**) Dynamic expression of miR-202-3p/5p in oocytes at different developmental stages. U6 was used as an internal control. Asterisk indicated significant difference (*p < *0.05).

In zebrafish, the pri-miR-202 encodes a pair of mature miRNAs, miR-202-5p and miR-202-3p (Fig. 1A). The tissue distribution patterns of miR-202-3p and -5p were examined by quantitative reverse transcription polymerase chain reaction (RT-PCR) in eight tissues including heart, liver, spleen, kidney, brain, muscle, testis and ovary. As shown in Fig. 1B, miR-202-3p was universally expressed in all examined tissues, and its expression level was comparatively high in heart, moderate in ovary, muscle and spleen, low in liver, testis, brain and kidney. However, miR-202-5p was specifically expressed in gonad, with a higher expression level in testis than in ovary. The dynamic expression patterns of miR-202-3p and -5p in oocytes at different developmental stages were further examined. As shown in Fig. 1C, miR-202-3p was almost undetectable in oocytes at stages I-III until stage IV, and the highest expression level was observed in mature oocytes. Whereas, the expression level of miR-202-5p in stage I and II oocytes was low, but the expression level gradually increased from stage III, and peaked in mature oocytes. In comparison with miR-202-3p, the expression of miR-202-5p was much higher and exclusive in mature oocytes.

### MiR-202-5p is a maternal miRNA in zebrafish

**Figure 2:**
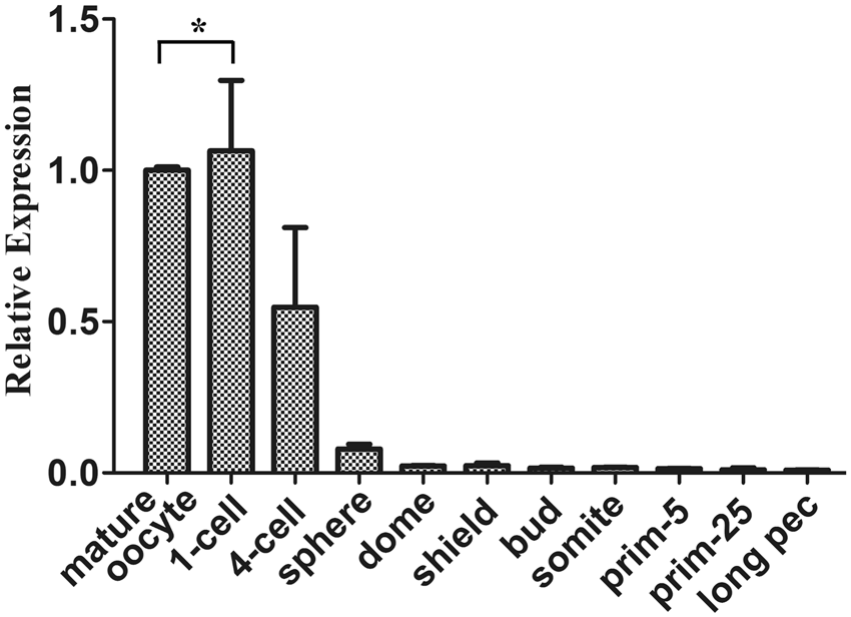
Temporal expression of miR-202-5p in mature oocytes and embryos at different development stages. U6 was used as an internal control. Asterisk indicated significant difference (*p < *0.05).

Subsequently, the embryonic expression pattern of miR-202-5p was examined by quantitative RT-PCR. As shown in Fig. 2, miR-202-5p was highly expressed in mature oocytes, and there was a slight up-regulation in 1-cell embryos after fertilization. During blastulation, the expression of miR-202-5p gradually decreased, and only very low expression was detected from gastrulation to long pec stage. These results indicated that miR-202-5p was a maternal miRNA.

### MiR-202-5p is a germ plasm-specific miRNA

**Figure 3:**
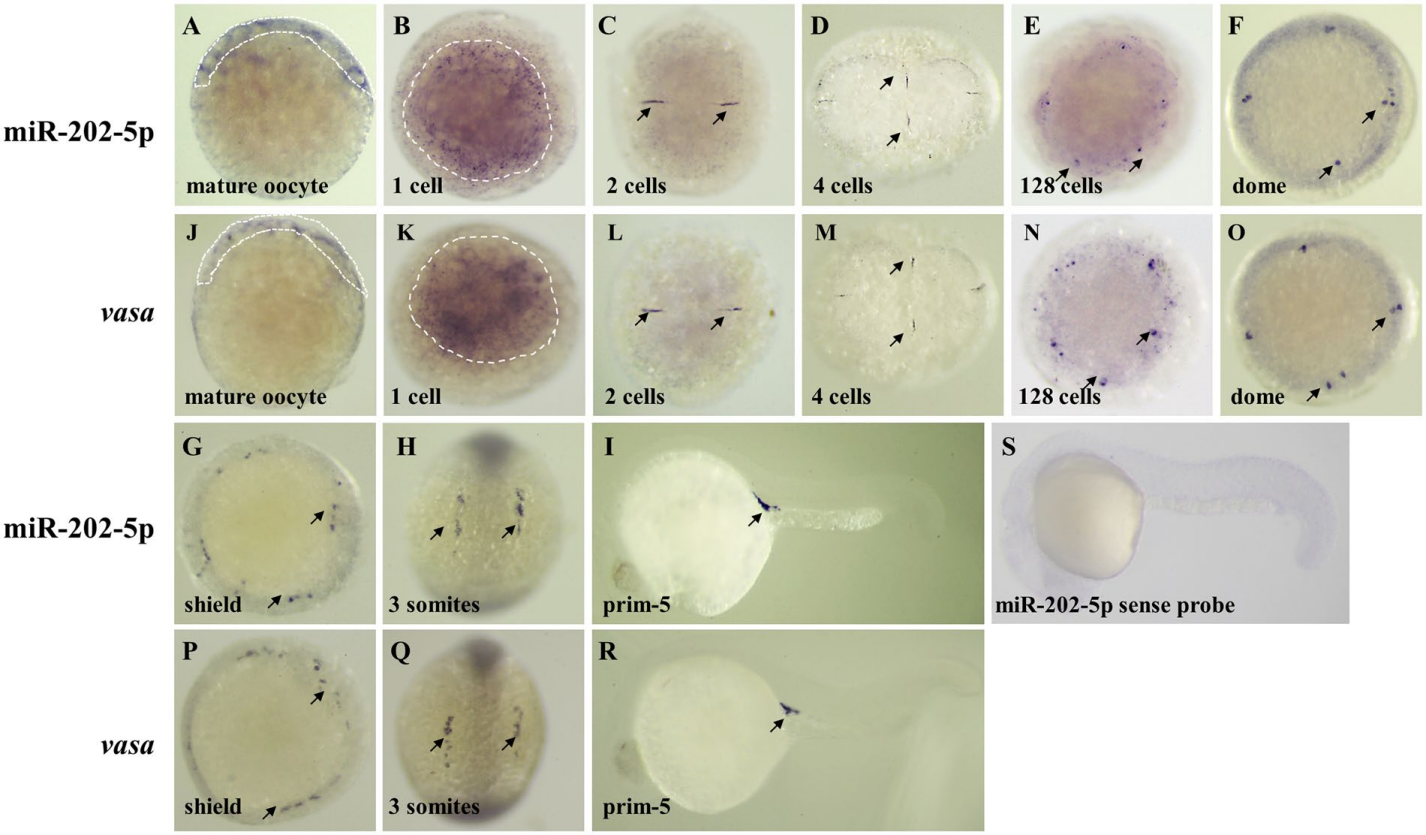
Spatial and temporal localization of miR-202-5p in mature oocytes and embryos at different development stages by WISH. A-I, localization of miR-202-5p; J-R, localization of *vasa* mRNA; S, negative control. The black arrows indicated the positive signals of miR-202-5p and *vasa* mRNA.

Furthermore, the spatial and temporal localization of miR-202-5p was revealed by WISH. As shown in Fig. 3A, miR-202-5p signals were present in a wide cortical band around the animal pole in mature oocytes. After fertilization, miR-202-5p signals were present in numerous granules throughout the cortex of 1-cell embryos (Fig. 3B). Subsequently, miR-202-5p signals were dominantly located at the cleavage furrows in embryos at 2 and 4 cells stages (Fig. 3C and D). At 128 cells stage, some granular miR-202-5p signals were detected at the animal pole (Fig. 3E). At dome stage, miR-202-5p-expressing cells were present at the four corners of the animal pole (Fig. 3F). At shield stage, miR-202-5p-expressing cells were distributed at the marginal region of the blastoderm (Fig. 3G). At 3 somites stage, miR-202-5p-expressing cells aggregated into two lines along the yolk syncytial layer (Fig. 3H). At prim-5 stage, miR-202-5p-expressing cells located in the genital ridge (Fig. 3I). This expression pattern of miR-202-5p was consistent to that of the known PGC marker *vasa* throughout early embryonic development (Fig. 3J–R). Meanwhile, we observed that miR-202-3p was maternally and universally expressed in almost all cells but not in PGCs during embryogenesis (Supplementary Fig. S1).

### Co-localization of miR-202-5p and *vasa* mRNA in germ plasm and PGCs

**Figure 4:**
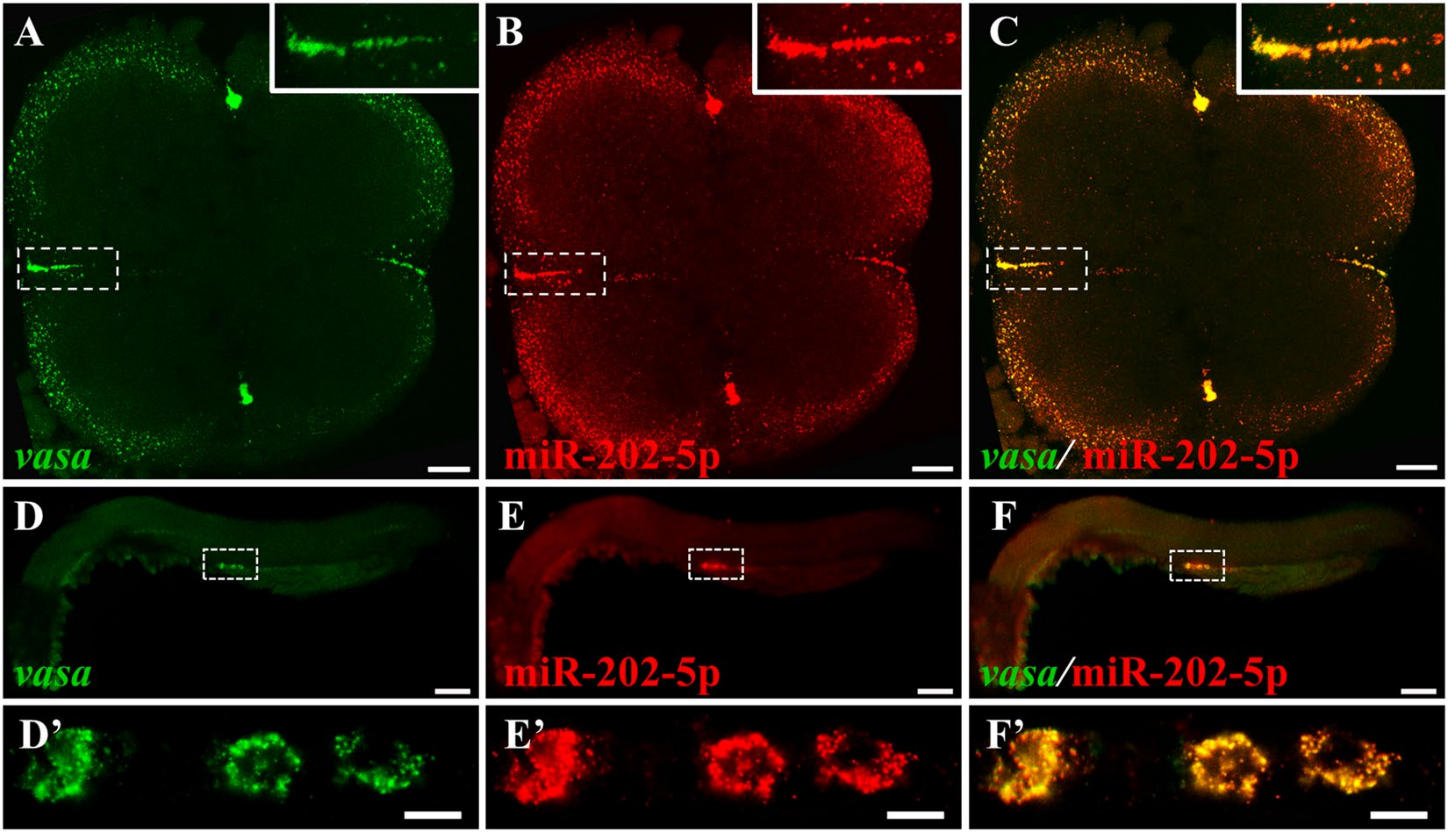
Co-localization of miR-202-5p and *vasa* mRNA by FISH. (**A** and **D**), *vasa* mRNA (green), (**B** and **E**) miR-202-5P (red); (**C** and **F**), merged. (**A**–**C**), embryo at 4-cell stage; D–F’, embryo at prim-5 stage. The smaller panels on top right of (**A**–**C**) indicated the high magnification of the white dotted areas, and D’–F’ are the high magnification of the white dotted areas in (**D**–**F**). Scale bars: 50 μm in (**A**–**C**), and 100 μm in (**D**–**F**).

To further demonstrate the specific distribution of miR-202-5p in germ plasm and PGCs, dual-color FISH was performed to examine the co-localization of miR-202-5p and *vasa* mRNA during embryogenesis (Fig. 4). At 4-cell stage, miR-202-5p and *vasa* mRNA co-localized at the cleavage furrows where the maternal germ plasm distributed (Fig. 4A–C). At prim-5 stage, miR-202-5p and *vasa* mRNA co-localized at the perinuclear germ granules of PGCs (Fig. 4D–F’). Therefore, similar to *vasa* mRNA, miR-202-5p was also a maternal germ plasm component, and specifically localized in zebrafish PGCs throughout embryogenesis.

## Discussion

In this study, we characterized the expression patterns of miR-202-5p and -3p. The expression patterns of these two miRNAs were quite different in tissues and embryos. Comparison to the universe expression of miR-202-3p, miR-202-5p was specifically expressed in gonads and was accumulated during oocyte maturation in female. Importantly, miR-202-5p was a maternal miRNA that was specifically expressed in germ plasm and subsequently in PGCs throughout embryogenesis. MiR-202-5p is the first miRNA identified in germ plasm and PGCs in zebrafish.

In vertebrates, pri-miR-202 encoded two opposing miRNAs, miR-202-3p and -5p. In mammals, miR-202-3p was not only involved in gonad development, but also identified as an important tumor repressor in inhibiting tumor progression^29–31^. It has been reported that miR-202-5p was highly expressed in Sertoli cells in embryonic mouse gonads^23^. In human, miR-202-5p was specifically localized in Sertoli cells and its expression significantly differed between fertile and sterile men, suggesting a novel interaction for regulating miRNA expression between the somatic and germ cell components of the seminiferous epithelium^24^. Therefore, the somatic cells expressing miR-202 regulated spermatogenesis in mammals. Differently, in lower vertebrates such as frog and zebrafish, miR-202 was expressed in germ cells. In frog, miR-202-5p was highly expressed in testis and was dramatically down-regulated in sterile males, suggesting a role of miR-202-5p in spermatogenesis^26^. Meanwhile, frog miR-202-5p was a gonad-specific miRNA and enriched in oocytes, indicating miR-202-5p also regulated oogenesis^25^. In zebrafish, dynamics of miRNA transcriptome showed that the expression of miR-202-3p and -5p were increased during oogenesis and spermatogenesis^27^. Our previous study revealed that miR-202-5p was highly expressed in spermatozoa and developing male germ cells at various stages in zebrafish^28^. Our present study demonstrated the different expression patterns between miR-202-5p and miR-202-3p in zebrafish, indicating the different functions of miR-202-3p and miR-202-5p. The low and universe expression of miR-202-3p in many tissues suggested a broad function. However, the gonad-specific expression pattern of miR-202-5p and its dynamic expression during oocyte maturation suggested its potential role in germ cell development. The high expression level of miR-202-5p in early embryos inherited from mature eggs and spermatozoa by fertilization strongly suggested that miR-202-5p might be an important regulator in PGC development.

So far, little is known about the embryonic expression pattern of miR-202 in lower vertebrates. In this study, RT-PCR examination revealed that miR-202-5p was maternally exist in unfertilized eggs, and there was a little up-regulation of miR-202-5p in 1-cell embryos, which might be due to an additional amount of miR-202-5p from spermatozoa upon fertilization. WISH and FISH detection further showed that miR-202-5p was localized in cortex around the animal pole in mature oocyte and was exclusively expressed in germ plasm and PGCs post fertilization. The decreased expression of miR-202-5p detected by RT-PCR was inconsistent with the increased number of miR-202-5p positive cells because of a lower proliferation rate of PGC than that of somatic cells. In zebrafish, miR-34 was the only reported maternal miRNA, which was involved in embryonic neurogenesis^32^. This study showed that miR-202-5p was the second maternal miRNA and it was the first germ plasm-specific miRNA in zebrafish. In animals with preformation mode, the specification of PGCs depends on the germ plasm, which comprises of various maternal proteins and RNAs. It had been found that germ plasm-specific long non-coding RNA *pgc* was important for transcriptional silence and chromatin structure^33, 34^. Since miRNA-mediated posttranscriptional regulation always led to gene silence, much attention was paid to miRNAs that might be involved in germ cell development. In animals with inductive mode, many miRNAs related to germ cell development had been identified and investigated. In mouse, the LIN28/let-7/BLIMP1 axis, in which RNA binding protein LIN28 negatively regulated let-7 to protect the expression of BLIMP1, was essential for PGC induction *in vitro* ^35, 36^. In human, the miR-372/let-7 axis regulated germ cell formation from embryonic stem cells^18^. In chicken, several PGC-enriched miRNAs were identified by microarray, and these miRNAs were highly expressed in male germ cells and even PGCs^17^. In preformation mode, although it had been demonstrated that miRNA pathways were important for germ cell development, no germ plasm-specific miRNA was identified^18^. Our present study showed that miR-202-5p was a novel germ plasm-specific miRNA in zebrafish. These results indicated the importance to further investigate the functions of miRNAs in germ cell development in animals with PGC preformation mode.

In present study we characterized the expression pattern of miR-202, and found that miR-202-5p was a novel germ plasm-specific miRNA in zebrafish. The unique expression pattern of miR-202-5p suggested that it might play an important role in PGC development in zebrafish, and further work was needed to investigate the function of miR-202-5p and the molecular mechanism by which miR-202-5p works.

## Methods

### Fish

Zebrafish, AB line wild type, were raised at 28 °C with 10 hours (h) darkness and 14 h light. All embryos were collected after natural spawning and staged as previous reported^37^. All procedures with zebrafish were approved by the Ethics Committee of Sun Yat-Sen University and the methods were carried out in accordance with the approved guidelines.

### Sample collection

Embryo samples were collected at different developmental stages including mature oocytes, and embryos at 1-cell stage, 4-cell stage, sphere, dome, shield, bud, somite stage, prim-5 (24 hours post fertilization, 24 hpf), prim-25 (36 hpf) and long pec (48 hpf) stages into Trizol reagent (Invitrogen) for RNA isolation. Embryos at above stages were also collected and fixed in 4% paraformaldehyde (PFA) in PBS at 4 °C overnight and stored in 100% methanol at −20 °C for WISH. Tissues including heart, liver, spleen, kidney, brain, muscle, ovary and testis, were collected from adult zebrafish into Trizol reagent for RNA isolation. Oocytes at different developmental stages were separated under a Leica stereomicroscope as previously described^38^.

### RNA isolation and RT-PCR

Total RNA was isolated by Trizol reagent (Invitrogen) according to the manufacturer’s instructions. The total RNA was reverse-transcripted using Mir-X™ miRNA Frist-Strand Synthesis Kit (Takara). The quantitative RT-PCR was performed in a Roche LightCycle 480 II (Roche) and SYBR® Premix Ex Taq™ II (Tli RNaseH Plus) as previously described^39^. The RT-PCR cycling conditions were: 94 °C for 2 min, followed by 40 cycles of 94 °C for 12 s, 60 °C for 12 s, 72 °C for 15 s. U6 was used as the internal control (The U6 primers were provide by Mir-X™ miRNA Frist-Strand Synthesis Kit). The relative gene expression was calculated with the 2^−ΔΔCT^ methods. Data were shown as mean ± SD from three independent experiments in triplicates. The primer sequences were listed in Table S1.

### Whole mount *in situ* hybridization (WISH)

The *vasa* cDNA sequence for synthesis of digoxin (DIG) or fluorescein isothiocyanate (FITC) labelled *vasa* sense and anti-sense RNA probes was as previously described^40^. The 1.1-kb *vasa* cDNA were cloned and sequenced by using a pair of *vasa* primers which containing a T7 core promoter at the 5′ end of the reverse primers. The primer sequences were listed in Table S1. The probes were synthesized by using DIG- or FITC-RNA labeling kit according to the manufacturer’s instructions (Roche). The sense and anti-sense DIG labelled RNA probes of miR-202-5p (Genbank accession number: MIMAT0003406) were synthesized by Ribobio (Guangzhou, China). WISH was performed as previously described^41^. Embryos were fixed in 4% PFA at 4 °C overnight, dehydrated in methanol at −20 °C overnight, rehydrated by a series of methanol/PBS gradient into 100% PBST, and treated with proteinase K and re-fixed in 4% PFA. Embryos were prehybridized with hybridization mix at 59 °C for 2-4 h, and hybridized with DIG-labelled miR-202-5p or *vasa* anti-sense probe at 59 °C overnight. After hybridization, embryos were washed in a series of SSCT gradient at 64 °C, and in PBST at room temperature. Subsequently, the embryos were blocked PBST with 2 mg/mL BSA and 2% normal goat serum for 2 hours at room temperature, and then incubated in alkaline phosphatase conjugated anti-DIG antibody (1:5000 diluted in blocking solution, Roche) at 4 °C overnight. Finally, the embryos were washed in PBST for 15 min six times, and the signal was detected using NBT/BCIP as the chromogenic substrate. The images were documented with a Leica stereomicroscope MZ10F equipped with a digital camera.

### Fluorescent *in situ* hybridization (FISH)

The dual-color FISH was performed as previously described^42^. The procedure before hybridization and washing steps were same to that of WISH. Differently, embryos were hybridized with DIG-labelled miR-202-5p and FITC-labelled *vasa* probe at 59 °C overnight. Embryos were washed in MABT (0.1 M Maleic acid, 0.15 M NaCl, 0.1% Tween-20, PH 7.5) for 10 min twice at room temperature before blocking, and blocked with 2% blocking reagent (Roche) in MABT for 2 h at room temperature, and then incubated in anti-fluorescein-POD sheep monoclonal antibody (1:2000 diluted in blocking solution, Roche) at 4 °C overnight. The signal was detected by using the TSA^TM^ Plus Fluorescence Systems according to the product manual (NEL756, NEN Life Science). FISH images were acquired with a Leica TCS-SP8 confocal microscope (Leica), and confocal Z-series image stacks collected at 1 μm intervals were assembled by LAS X basic software.

### Statistics analysis

All statistics were calculated using SPSS version 20. Differences between control and treatment groups were assessed by one-way ANOVA. *p < *0.05 was considered to be statistically significant.

## Electronic supplementary material

Supplementary information
