# Supplementary material for: MiR-202-5p is a novel germ plasm-specific microRNA in zebrafish
Source: Sci Rep. 2017 Aug 1;7:7055. doi: 10.1038/s41598-017-07675-x (PMC5539161; doi:10.1038/s41598-017-07675-x)

MiR-202-5p is a novel germ plasm-specific microRNA in zebrafish

Jing Zhang[1], Wei Liu[1], Yilin Jin, Peng Jia, Kuntong Jia[*] & Meisheng Yi[*]

Guangdong Provincial Key Laboratory of Marine Resources and Coastal Engineering, Zhuhai Key Laboratory of Marine Bioresources and Environment, School of Marine Sciences, Sun Yat-sen University, Guangdong, China.

[1] These authors contributed equally to this work.

*Correspondence and requests for materials should be addressed to M. Y. (yimsh@mail.sysu.edu.cn) or K. J. (jiakt3@mail.sysu.edu.cn)

Supplementary Information: Table S1 and Figure S1

Supplementary Table S1. Primer sequences

Name	Sequence (5’-3’)
miR-202-5p	TTCCATGCATATACCTCTTTG
miR-202-3p	AGAGGCATAGGGCATGGAAAA
vasa F	AGATCTCAACCACAAGCATCCA
vasa R	TAATACGACTCACTATATGTTACAGTCAGTCACT

The T7 core promoter sequence in *vasa* reverse (*vasa* R) primer was underlined

Supplementary Figure S1. Embryonic expression patterns of miR-202-3p. WISH detection of miR-202-3p expression in embryos at different developmental stages (A) 1-cell stage; (B) 4-cell stage; (C) dome stage; (D) 3-somite and (E) prim-5 stage.

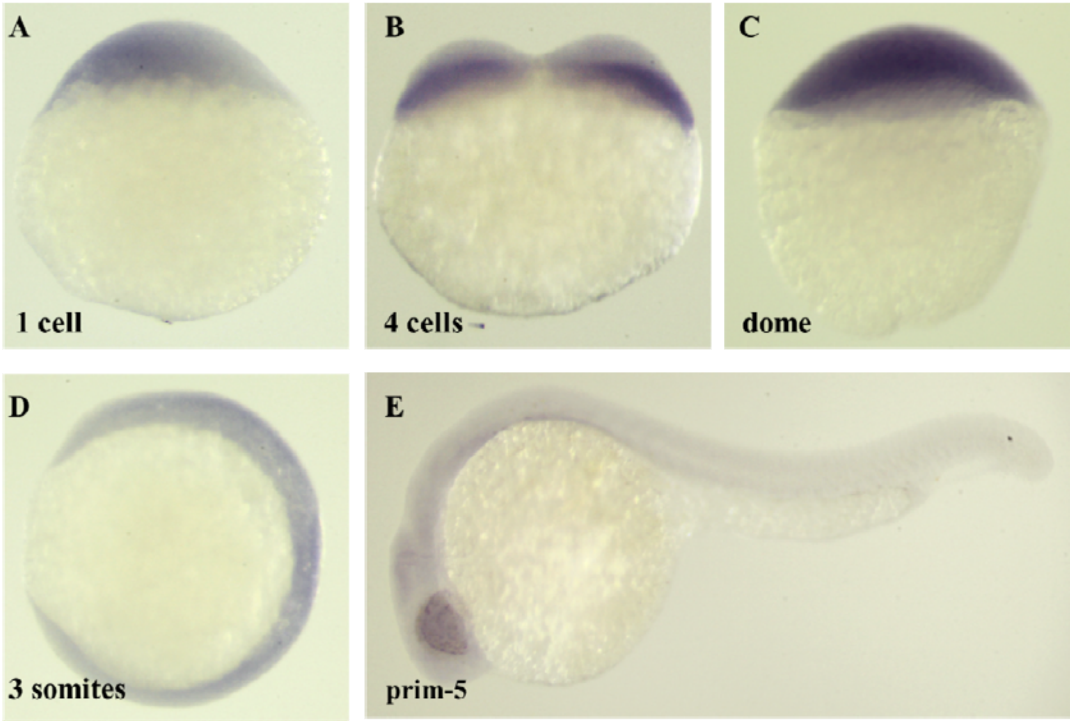

Supplement: Supplementary file 1 — Supplementary information [file 41598_2017_7675_MOESM1_ESM.pdf]
